# Voluntary Medical Incident Reporting Tool to Improve Physician Reporting of Medical Errors in an Emergency Department

**DOI:** 10.5811/westjem.2015.8.27390

**Published:** 2015-12-08

**Authors:** Nnaemeka G. Okafor, Pratik B. Doshi, Sara K. Miller, James J. McCarthy, Nathan R. Hoot, Bryan F. Darger, Roberto C. Benitez, Yashwant G. Chathampally

**Affiliations:** University of Texas Health Science Center, Department of Emergency Medicine, Houston, Texas

## Abstract

**Introduction:**

Medical errors are frequently under-reported, yet their appropriate analysis, coupled with remediation, is essential for continuous quality improvement. The emergency department (ED) is recognized as a complex and chaotic environment prone to errors. In this paper, we describe the design and implementation of a web-based ED-specific incident reporting system using an iterative process.

**Methods:**

A web-based, password-protected tool was developed by members of a quality assurance committee for ED providers to report incidents that they believe could impact patient safety.

**Results:**

The utilization of this system in one residency program with two academic sites resulted in an increase from 81 reported incidents in 2009, the first year of use, to 561 reported incidents in 2012. This is an increase in rate of reported events from 0.07% of all ED visits to 0.44% of all ED visits. In 2012, faculty reported 60% of all incidents, while residents and midlevel providers reported 24% and 16% respectively. The most commonly reported incidents were delays in care and management concerns.

**Conclusion:**

Error reporting frequency can be dramatically improved by using a web-based, user-friendly, voluntary, and non-punitive reporting system.

## INTRODUCTION

The emergency department (ED) is an error prone environment, with previous studies reporting 51–70% of errors occurring in the ED as preventable.^1^–^3^ This proportion is higher than any other patient care area in those studies. ED clinicians manage multiple patients in a complex-chaotic environment. High cognitive loads and frequent interruptions have been reported as fundamental sources of medical error in the ED.^2^,^4^,^5^ In regard to crowding,^6^,^7^ the ED remains an extremely decision-dense environment, where competing interests must be frequently re-prioritized according to continuously changing conditions. Identifying and remediating these errors, as well as mitigating harm, have become fundamental operational imperatives of ED leadership.

Medical errors must be accurately identified and contextualized in order to appropriately analyze them and create prevention strategies.^8^ Incident reporting systems are valuable systems to aid in the identification of errors.^9^–^12^ However, most are institution-based and centrally managed by the risk management and quality assurance (QA) departments of the hospital, rather than the departments in which the error occurred.^13^,^14^ Lack of service line ownership, along with burdensome reporting processes and fear of liability and embarrassment^15^ have been described as the reasons for extremely low physician incident reporting rates.^13^,^16^

In 2008, our institution’s emergency medicine (EM) QA committee analyzed the existing process of error evaluation and discovered that the process was ineffective and inefficient. Very few ED errors were voluntarily reported to the hospital-wide (centralized) system and those reported often lacked sufficient detail for meaningful analysis. The majority of errors were identified based on inconsistent and non-standardized referrals from other services or electronic medical record (EMR) reviews using the following triggers: (1) unscheduled return to the ED within 72 hours of initial visit with admission/hospital observation; (2) death in the ED; (3) death within 72 hours of admission; and (4) an unanticipated escalation of care (“rapid response”) within 24 hours of admission. The yield of actionable errors from other service lines and triggered reviews was poor. Both methods required extensive EMR review to identify involved personnel and determine which details were relevant. Accounts obtained from the involved clinicians regarding patient encounters were non-standardized, often incomplete, and subject to recall bias due to delays of 30 days or more from the date of the incident.

A more timely and efficient method to identify errors that occur in the ED was necessary in order to create an actionable medical error registry. Therefore, as part of a quality improvement strategy, a plan was developed to create a registry that would facilitate continuous analysis of ED errors. The objective of the analysis was to identify the system, cognitive and non-remediable factors that contributed to the error in order to make recommendations to prevent the recurrence of the error. Increasing the reporting of larger proportion of all potential errors was critical to the development of a robust error registry. The QA committee was comprised primarily of emergency physicians certified by the Physician Quality and Safety Academy, an intra-institutional program designed to educate physicians in the application of improvement science methods and strategies to attain departmental and institutional quality goals. In this article, we describe how a service line specific, voluntary, incident reporting system was created and used to improve emergency provider reporting in a peer-review protected environment. The focus of this article is on the development of the incident reporting system and its effect on reporting, rather than on the incident review process. To our knowledge, there are no publications that describe mechanisms that increased ED physician incident reporting.

## METHODS

The reporting system was developed in an EM residency training program with two sites, an urban tertiary referral, Level I trauma center with an annual census of 60,000 ED patient visits, and a county hospital with an annual census of 70,000 ED patient visits. The incident reporting system was based on the characteristics described by Dr Leape,^17^ and used the characteristics described for a successful incident reporting system. These included being voluntary, simple to use, non-punitive, confidential, timely, responsive, and system oriented.

The first phase of the development was designed to address the inefficiencies of the prior system, adopt characteristics of successful error reporting systems, and remove the reporting barriers outlined in the literature.^17^–^21^ The system operated independently of the hospital-wide reporting systems at both EDs in order to ensure that the EM QA committee maintained the ability to manage reported data. Access to the system was limited to EM faculty, residents and advanced practice providers (APP). The rationale for this limitation was to ensure that the initial focus was on those participants that the literature identified as poor reporters. All iterations of the system were web-based and password protected, accessible from any location with Internet access. Data entry was limited to a single page and standardized for listed incident types. The required fields were related to patient and clinician identification, selection of a predefined incident types and a free text narrative. We limited the incident types to seven general categories familiar to reporters and easily identifiable to those without quality science expertise, specifically: management concern, delay in care, procedural issue, medication error, handoff-checkout issue, near miss, consultation issue, and diagnostic error. The system limited users to one incident type per report; however, there was no limit on the number of involved clinicians that could be selected.

All reported data were stored on a password-protected database accessible solely by the QA committee members. Each incident reporter was identified using the username and password required for access and that information was displayed only to the EM QA committee. The rationale for mandatory reporter identification to the committee was to ensure that reporters submitting unclear or incomplete reports could be contacted for clarification. All clinicians involved with an incident report were contacted by email and asked to complete a standard paper form that included sections for the following: (1) a free text narrative of the patient encounter, (2) selection of predefined contributing factors (system, cognitive, and non-remediable), (3) their impression of the patient acuity during their evaluation. as well as (4) their impression of state of the department before, during and after the patient encounter. The committee used the involved clinicians’ impression of the patient acuity and state of the department to gain a better appreciation of the environmental context in which the clinical decisions were made. Only the EM QA committee members had access to the details of the involved clinician reports. The multidisciplinary EM QA committee, made up of both quality science and clinical domain experts including physicians, APPs, and nurses, reviewed the reported incidents and provided feedback to the involved and reporting clinicians either directly or during the monthly QA conference. In an attempt to mitigate the negative associations of incident reporting as only representing errors, “Interesting case” and “Resident/APP excellence” menu items were added as new incident types in August 2010.

The second major iteration of the system included email notification of all identified involved clinicians immediately following the initial incident report submission, based on the rationale that by decreasing turnaround time from incident occurrence to clinician notification would decrease clinician recall bias. Involved clinicians, following such notification, could read the submitted incident narrative without knowledge of the reporter’s identity. Clinicians were able to renounce involvement in the incident with a single click or recount their perspective of the patient encounter using a free-text narrative and checkboxes. They were also required to select from the predefined system, cognitive and non-remediable factors that contributed to the error using checkboxes. Repeat email notifications were sent to the involved clinician if they did not respond within 72 hours of incident submission. Each clinician could review the list of incidents that they reported as well as those in which they were listed as the involved clinician. The summary of the QA incident review along with suggestions to decrease the recurrence and harm of the incident could be viewed from that list. This last feature allowed the QA committee to provide timely, direct and individualized feedback to the involved as well as the reporting clinicians. The QA committee also had the ability to remove incorrectly assigned clinicians as well as assign new clinicians identified during the incident review. Critical care cases, documentation issue, triage issue and boarding issue were added as new incident types, and the ED clinical pharmacist was given access to report incidents. All the iterative modifications of the system were based upon the feedback provided by the users and agreed upon by all members of the QA committee.

Formal review of reported incidents by the EM QA committee occurred each month. Monthly cases were anonymously presented to residents, APPs, faculty physicians and nursing leadership to discuss errors with educational merit, disseminate policy or system modifications, generate consensus on best practices, and reinforce commitment to patient safety. Objectives of the case presentations were to encourage clinicians to (1) recognize the error prone nature of the ED, (2) report near misses, errors, and adverse events without fear of negative repercussions, (3) increase collaboration with other ED staff and medical disciplines to seek out and sustain safer workflows and processes, and (4) provide feedback of the errors identified in the ED.

This project was approved by the institutional Committee for the Protection of Human Subjects. It was part of a QA/process improvement strategy to decrease the recurrence of medical errors in the ED.

## RESULTS

**Figure 1:**
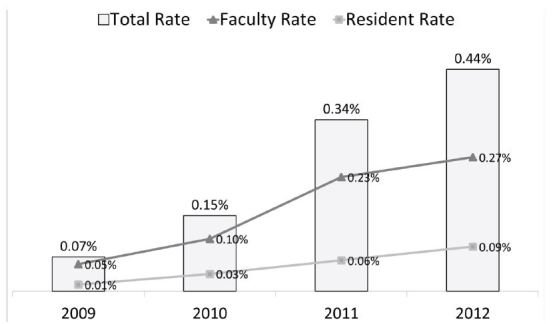
Annual total rate of all incidents reported with faculty and trainee rates of incident reporting.

Between March 2009 and December 2012, 1,229 incidents were reported. The total incident reports for 2009, 2010, 2011 and 2012 were 81, 177, 410 and 561 respectively. When compared to the total ED visits over this time period, the rate of reported incidents were 0.07%, 0.15%, 0.34% and 0.44% for fiscal year 2009 to 2012 respectively (Figure 1). Incident reporting at the tertiary care ED steadily increased over the years; however, a four-fold increase in incident reporting was noted at the county ED after the second quarter in 2011.

**Figure 2:**
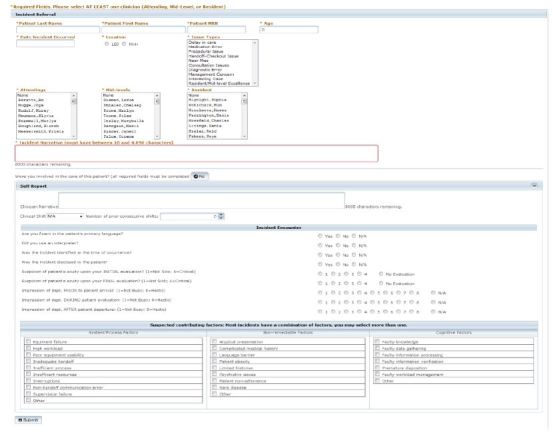
Screen shot of the data entry section of latest iteration of the medical incident reporting system. The attending, midlevel and resident names in this image are fictitious. Any resemblance to real persons, living or dead, is purely coincidental.

**Figure 3:**
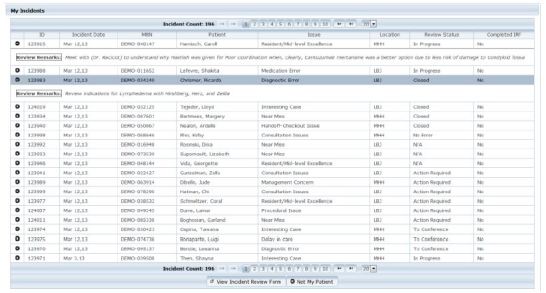
Screen shot of the incident review section of latest iteration of the medical incident reporting system. The “Self Report” section of the data entry section is only displayed if the reporter indicates involvement in the incident. The review remarks in the incident review section contain the feedback from the EM QA committee for each incident and are only displayed by user selection. Medical record numbers and patient names in the adjacent image are fictitious. Any resemblance to real persons, living or dead, is purely coincidental.

Figures 2 and 3 illustrate the data entry and review sections of the latest version of the medical incident-reporting system. Table shows the number of distinct ED faculty, resident and APP reporters at both EDs as well as their relative percentage of available ED clinicians that year. With the exception of the APPs at the tertiary ED, the number of faculty, resident and APP reporters increased each year at both EDs. The number of resident reporters at the county ED dramatically increased in 2012.

Increased faculty participation was demonstrated every year; 33% of faculty reported an incident in 2009 while 76% of faculty reported an incident in 2012. Similar results were noted with resident and APP participation. Resident participation increased from 24% of residents reporting an incident in 2009 to 72% reporting an incident in 2012, and APP participation increased from 4% of midlevel clinicians reporting an incident in 2009 to 61% reporting an incident in 2012.

Faculty submitted the most incident reports each year at both EDs (Figure 1). Generally the number of reports by each reporter type at both EDs increased each year. The most significant increase in faculty and APPs reporting occurred in 2011 at the county ED.

The average number of reports per distinct reporter increased each year at both hospital sites with the exception of the faculty physician reports at the tertiary care ED in 2012.The most commonly reported incidents each year were management concern and delay in care.

## DISCUSSION

We have described the successful design, implementation and utilization of a department-specific incident reporting system, a core component of a comprehensive quality improvement process. The frequency of incident reporting by physicians increased from 81 reported incidents in 2009 to 561 in 2012. To provide perspective as to the number of incidents reported, our tertiary care institution, which is an 800-bed hospital, receives approximately 1,000 incidents/year reported by physicians, which includes all the departments of the hospital, in their central variance reporting system. With the creation of our department-specific tool as part of our comprehensive quality improvement program, we generated 50% of those reports as a single department.

We believe that the increase in reported incidents also represents a shift in the safety culture of the department, as increasing comfort with reporting is becoming a part of the routine ED operation. In addition, this system has allowed a more complete assessment of reported incidents by providing more comprehensive data regarding the encounter, allowing for a more accurate understanding of factors that may have played a role. Despite the increase in reported events and better understanding of the involved factors in the potential error, it is very difficult to assess the impact of these processes on patient outcomes due to the fact that it is difficult to measure the number of subsequent patient encounters with similar clinical presentation that were managed in a more appropriate manner. Anecdotal evidence from our reported incident reviews suggests findings similar to other published studies.^22^,^23^ For example, our trigger-based EMR review process was inefficient and ineffective for the detection of near misses, medication errors and procedure errors. Even though the majority of errors identified via the traditional trigger-based EMR chart review were treatment delays, diagnostic errors and inappropriate dispositions, higher proportions were noted with our reporting system.

This project also corroborates the findings of previous studies,^13^, ^24^ showing that trainees did not report incidents as frequently as the faculty. However, in our study the rate of reporting for the trainees improved in similar fashion to the faculty reporting. This is an important finding as it reiterates the importance of adequate curriculum development and education in the area of practice-based learning and system-based practice core competencies, which have been part of the evaluation of U.S. trainees for the past decade. We incorporated the data from reported incidents into performance improvement projects for the trainees and faculty, required for trainees by the Accreditation Council for Graduate Medical Education and for faculty as part of their re-certification process.

In addition to being consistent with prior publications that described the characteristics of a successful reporting systems and suggested methods to remove barriers to physician reporting,^17^,^18^,^21^,^25^ our project suggests other characteristics necessary to improve reporting and subsequent review. Our iterative developmental process with active feedback solicitation suggested that a successful system must do the following: (1) provide immediate notification to the involved clinicians to decrease the recall bias, (2) allow the involved clinicians to enter their narrative of the patient encounter directly into the system for a timely incident review, (3) allow the involved clinicians to receive timely, direct and individualized feedback from the system regarding incidents they were either involved in or reported to ensure that accessing the system becomes habitual, (4) include non-medical error incident types to the reporting system to mitigate negative connotations associated with use of the system, (5) allow for analysis of the reported incidents (at least initially) by peer experts who understand the environmental context in which the incident occurred, and (6) allow for restructuring of the incident review process and workflow as reporting increases is necessary to ensure timely feedback.

To our knowledge, this is the first report describing a service specific incident reporting tool as part of a comprehensive quality improvement process to improve physician reporting and subsequent analysis of the incidents to better ascertain the involved factors in the occurrence of errors.^26^ We have also provided a potential plan for other departments attempting to create a similar system at their individual institutions.

## LIMITATIONS

The first and most important limitation is the inability to determine a true denominator in order to measure error reporting rates. However, we have provided a rate of incident reports as compared to total ED visits for the respective years to further clarify that the reported incidents did truly represent an increase in incident reporting. Second, differentiating the portion of the reported incidents resulting from an adoption of a safety culture from the portion resulting from access to an improved reporting system is difficult. In addition, in this type of work it is very difficult to delineate the true impact on patient outcomes of increased incident reporting. However, without an appropriate mechanism for reporting potential errors, not even the best intended safety systems can increase the identification, analysis and remediation of errors. Lastly, we understand that due to the voluntary nature of such systems, this process of error identification is subject to selection bias and likely represents only a fraction of existing errors.

## CONCLUSION

Our project illustrates that error reporting frequency by physicians can be dramatically improved by using a web-based, user-friendly, voluntary, and non-punitive reporting system. To be successful, such a system must evolve to meet the requirements of users. Our study also suggests that transparent and decentralized service-specific incident review and quality improvement teams could support error-reduction strategies for the hospital system by increasing incident reporting, analysis, and interventions within specific service lines.

Despite the inability to capture all errors, these reported incidents represent an important opportunity for improving patient safety, and can serve as an foundation for improvement in the education of our trainees in the areas of practice-based learning and system-based practice, as well as in creating performance improvement projects required for re-certification of the faculty. This project is a critical component of transforming the departmental culture into one that is patient centered, self-reflective, and proactive regarding practice improvement.

## Figures and Tables

**Table:** Number for distinct reporters per reporter type and the relative percentage of emergency department (ED) clinical staff.

	Tertiary ED			County ED		
	
Year	Faculty	Resident	Midlevel	Faculty	Resident	Midlevel
2009	12/37 (32%)	11/43 (24%)	0/5 (0%)	8/37 (22%)	0/9 (0%)	1/23 (4%)
2010	19/45 (42%)	22/45 (49%)	2/7 (29%)	12/42 (29%)	3/32 (9%)	6/21 (29%)
2011	23/42 (53%)	26/53 (49%)	1/10 (0%)	24/42 (57%)	6/53 (11%)	13/22 (59%)
2012	29/41 (71%)	31/60 (52%)	0/16 (0%)	26/38 (68%)	29/60 (48%)	14/23 (61%)
